# Software and hardware infrastructure for research in electrophysiology

**DOI:** 10.3389/fninf.2014.00020

**Published:** 2014-03-07

**Authors:** Roman Mouček, Petr Ježek, Lukáš Vařeka, Tomáš Řondík, Petr Brůha, Václav Papež, Pavel Mautner, Jiří Novotný, Tomáš Prokop, Jan Štěbeták

**Affiliations:** ^1^Department of Computer Science and Engineering, Faculty of Applied Sciences, University of West BohemiaPlzeň, Czech Republic; ^2^New Technologies for the Information Society, Faculty of Applied Sciences, University of West BohemiaPlzeň, Czech Republic

**Keywords:** electrophysiology, event related potentials, infrastructure, neuroinformatics, workflow, portal, signal processing methods, stimulator

## Abstract

As in other areas of experimental science, operation of electrophysiological laboratory, design and performance of electrophysiological experiments, collection, storage and sharing of experimental data and metadata, analysis and interpretation of these data, and publication of results are time consuming activities. If these activities are well organized and supported by a suitable infrastructure, work efficiency of researchers increases significantly. This article deals with the main concepts, design, and development of software and hardware infrastructure for research in electrophysiology. The described infrastructure has been primarily developed for the needs of neuroinformatics laboratory at the University of West Bohemia, the Czech Republic. However, from the beginning it has been also designed and developed to be open and applicable in laboratories that do similar research. After introducing the laboratory and the whole architectural concept the individual parts of the infrastructure are described. The central element of the software infrastructure is a web-based portal that enables community researchers to store, share, download and search data and metadata from electrophysiological experiments. The data model, domain ontology and usage of semantic web languages and technologies are described. Current data publication policy used in the portal is briefly introduced. The registration of the portal within Neuroscience Information Framework is described. Then the methods used for processing of electrophysiological signals are presented. The specific modifications of these methods introduced by laboratory researches are summarized; the methods are organized into a laboratory workflow. Other parts of the software infrastructure include mobile and offline solutions for data/metadata storing and a hardware stimulator communicating with an EEG amplifier and recording software.

## 1. Introduction

As in other areas of experimental science, operation of electrophysiological laboratory, design and performance of electrophysiological experiments, collection, storage and sharing of experimental data and metadata, analysis and interpretation of these data, and publication of results are time consuming activities. If these activities are well organized and supported by a suitable infrastructure, work efficiency of researchers increases significantly.

Our research group, a member of the Czech National Node of International Neuroinformatics Coordinating Facility (INCF, 2013), focuses on research of brain electrical activity using the methods and techniques of electroencephalography (EEG) and event related potentials (ERP). Our neuroinformatics laboratory, which started to operate in 2005, is currently equipped with a number of commercial and custom hardware devices and software tools. Besides the basic electrophysiological infrastructure (amplifier, synchronization device, recording and analytic software, and software for presentation of stimuli) the laboratory equipment includes a sound and electrically shielded booth, a car simulator including a car cockpit, wheel and pedals connected to the computer, projector, and software tools for the simulation of driving environment and driving itself. Since the group has been solving difficulties with the laboratory operation (software and hardware tools and the whole infrastructure) from the beginning of its research activities, this paper introduces not only the current state of the laboratory infrastructure but also some essential intermediate steps in its building. The presented infrastructure is also more oriented to the processing of data from ERP than EEG experiments; as a result e.g., the methods for ERP component detection are highlighted in the text.

The paper is organized in the following way. The section Materials and Methods contains the description of the state of the art in building infrastructures for research in electrophysiology and neurophysiology. The next subsections first introduce the whole concept of the laboratory infrastructure; then some infrastructural parts are described. The section Results provides information about the current state and some implementation details of the selected parts of the infrastructure. The section Discussion mainly discusses the potential limitations of the built infrastructure and primarily speculates on the future direction of the proposed infrastructure.

## 2. Materials and methods

### 2.1. State of the art

Building large infrastructures for research has become very popular with the rapid development of computers and hardware devices, programming languages and technologies, software tools, and online communication. This development has also spread to neuroscience and neuroinformatics to support the efficiency of the research in the field.

Coordinating activities in neuroinformatics are led by INCF that develops and maintains database and computational infrastructure for neuroscientists. Software tools and standards for the international neuroinformatics community are being developed through the INCF Programs, which address infrastructural issues of high importance to the neuroscience community (INCF, 2013). To enable collaboration between researchers through the sharing of neuroscience data, INCF introduced the INCF Dataspace (INCF Group, 2013). It associates INCF nodes data sources in a distributed system based on iRods solution. Technically, data are managed locally by individual nodes and connected using catalog servers. From a user perspective it works as a large data file system accessed through a web interface. In other words, all these zone servers, connected resources, and hosted datasets build a distributed network of shared data.

In electrophysiology (and in neurophysiology in broader sense) we can also find activities focusing on building larger software and/or hardware infrastructures. These activities, carried out by universities, research institutions and private companies, include cooperation of various hardware devices supported by related software tools in laboratories, definition of data formats, solutions for storing, managing, and sharing data and metadata, and development of methods and workflows for data processing, visualization and interpretation. Finally, more complex, usually web based solutions then can serve as virtual laboratories. The following parts of this section introduce some of the various approaches and activities that contribute to building infrastructures in electrophysiology (neurophysiology). More complex and already existing infrastructures are also mentioned.

The description of the electrophysiological domain (and description of any domain in general) could be provided at different levels of abstraction and includes both cooperating and competing techniques and approaches (e.g., classical data modeling vs. ontological modeling). Moreover, various physical repositories are used to store domain data and metadata. Then various programing languages, coding and architectural styles, technologies, and software tools are used to process these data and metadata. Since it is out of scope of this paper to focus on and describe the differences and relationships between various techniques and approaches, the following selection just introduces well known approaches and activities.

Open Metadata Markup Language (odML) (Grewe et al., 2011) is a flexible and unified metadata format for annotation data in neurophysiology. This language defines terminologies for the domain, but simultaneously is extensible and flexible for science that continually changes, and does not restrict the user by requiring entries. It increases its potential to become an exchange/sharing format for electrophysiology data. Metadata stored in odML are linked to the related data, for which a suitable exchange/sharing format is also looked for. Currently, great deal of attention is paid to HDF5 (Hierarchical Data Format) (HDF Group, 2013), or similar formats based on HDF5 (e.g., epHDF). HDF5 is a data model, library, and file format for storing and managing data. It supports an unlimited variety of data types, and is designed for flexible and efficient I/O and for high volume and complex data. NoSQL document databases, due to their flexibility, are also very promising for long term storage of electrophysiological data and metadata. We tested StorageBIT (Carreiras et al., 2013) that combines HDF5 and MongoDB. HDF5 ensures data persistency while MongoDB is a front end for data access. Our tests (Jezek et al., 2014) proves that MongoDB is equivalent to relational databases from the performance point of view. Moreover it provides a better flexibility.

One of the leading initiatives for data sharing is the Neuroscience Information Framework (NIF, 2013) as a dynamic inventory of registered web-based neuroscience resources (data, materials, and tools). NIF enables access to public research data and tools through an open source environment (Gardner et al., 2008). Currently, it is with more than 6,400 resources one of the largest collection of neuroscience data. Each new resource has to pass at least one of three levels of registration. These levels specify depth of resource integration into NIF; level 1 provides information about the resource, level 2 provides direct access to resource's web services, and finally, the resource is sustained by an ontology at level 3. G-Node data management platform is a sharing facility that allows data organization, annotation, access and sharing. All can be managed via web-based interface as well as via RESTful API; access using an external application is possible. G-Node provides Matlab and Python scripts clients (G-Node, 2013).

In addition to a proper data and metadata format, ontologies are also helpful for data sharing. Significant representatives of bio-ontologies dealing with neurophysiology and electrophysiology are Neural Electro Magnetic Ontologies NEMO (Dou et al., 2007) and the Ontology for Biomedical Investigations OBI (Brinkman et al., 2010). The ontology built within the NEMO project provides formal semantic definitions of concepts in ERP research, including ERP patterns, spatial, temporal, functional (cognitive/behavioral) attributes of these patterns, data acquisition and analysis methods (Dou et al., 2007). OBI is an ontology for biological and clinical investigation description. Its terminology contains domain-specific terms and universal terms for general biological and technical usage. Finally, the ontology will represent the design of an investigation, the protocols and instrumentation used, the material used, the data generated and the type analysis performed on it (Brinkman et al., 2010).

Methods, techniques and tools for ERP signal processing are also a very important part of the software infrastructure in electrophysiology. The standard approach for event-related potential (ERP) signal processing can be divided into following steps: analog to digital conversion, filtering, segmentation, latency correction, averaging, and methods for detection and analysis of ERP components. As a result, a set of parameters describing ERP components is obtained. From this result, useful information about the medical condition of the measured subject can be determined. (Picton et al., 1995; Luck, 2005). Proven techniques for ERP signal processing include wavelet transform (Quiroga and Garcia, 2003), matching pursuit (Aviyente et al., 2006), Independent Component Analysis (ICA) (Makeig et al., 1997), Principal Component Analysis (PCA) (Dien, 2012), and Hilbert-Huang transform (HHT) (Cong et al., 2009). For subsequent classification, Linear Discriminant Analysis (LDA), Support Vector Machines (SVM), and multi-layer perceptron are among the most frequently used methods (Lotte et al., 2007).

Different signal processing tools have been used for event-related data potential data processing within neuroinformatics community. Matlab (MATLAB, 2012) is the most popular since it is easy to use and implements many signal processing methods—either in its core (e.g., temporal filtering, FFT), or in default toolboxes (wavelet transform, matching pursuit, etc.). Furthermore, EEGLAB (Delorme and Makeig, 2004) can be directly used for the analysis of EEG/ERP experiments. EEGLAB is an interactive Matlab toolbox for continuous and event-related neurophysiological data processing. It allows researchers to load data in various formats, to extract epochs using stimuli markers, to remove artifacts (e.g., by using ICA), etc. The BrainVision Analyzer (BrainProducts, 2012) is a complex tool for neurophysiological data analysis. It provides an easy-to-use user interface, multiple import and export features, different views for visualization, and methods for signal processing and analysis. EEGVIS (Robbins, 2012) is a MATLAB toolbox that allows users to quickly explore multi-channel EEG and other large array-based data sets using multi-scale drill-down techniques. This toolbox can be used directly in MATLAB at any stage in a processing pipeline, as a plug-in for EEGLAB, or as a standalone precompiled application without MATLAB running.

The CARMEN project (Carmen, 2013) is an effort to create a virtual laboratory. It allows neuroscientists to share and exploit data, programs (services) and expertise from neurophysiological experiments. Neural activity recordings (signals and image series) are the primary data types. The CARMEN Portal is a web interface onto the CARMEN system accessed via a standard web browser that provides users with access to the computer and data storage resources. The project also developed a workflow generation and execution system within the platform. The Java-based CARMEN Workflow Tool consists of a graphical design tool, a workflow engine, and access to a library of CARMEN services and common workflow tasks. It supports both data and control flow, and allows parallel execution of services (Carmen, 2013).

Several initiatives and/or pilot studies also try to provide a solution for researchers to efficiently work out of laboratories using portable devices as laptops, tablets or mobile phones. Clinician Assessment and Remote Administration Tablet (CARAT) (Turner et al., 2011) is a Microsoft Windows tablet adapted to collect and administer clinical assessments in large scale demographic or neuropsychiatric studies. It uses an architecture with two modules. The first one set-ups the clinical study while the second one serves to data collection. Collected data are synchronized with a remote database. Research Electronic Data Capture (Harris, 2012) (REDCap) is a software application and workflow methodology designed to collect and manage data for research studies. REDCap Mobile (Borlawsky et al., 2011) is a solution that describes encrypted laptops with a push-pull relationship to the centralized REDCap database to allow data collection while off-line. Such solution is suitable in studies that need to be performed on places without an internet access as hospitals or jails.

Devices for presenting and synchronizing stimuli and responses to them are also an important part of the infrastructure in electrophysiology. Hardware and software stimulators are produced and sold by multinational companies Metrovision, LKC Technologies, Grass Technologies, Inomed and Neurobehavioral systems. Their production is usually very specific and intended for medical purposes.

### 2.2. Overall architecture

**Figure 1:**
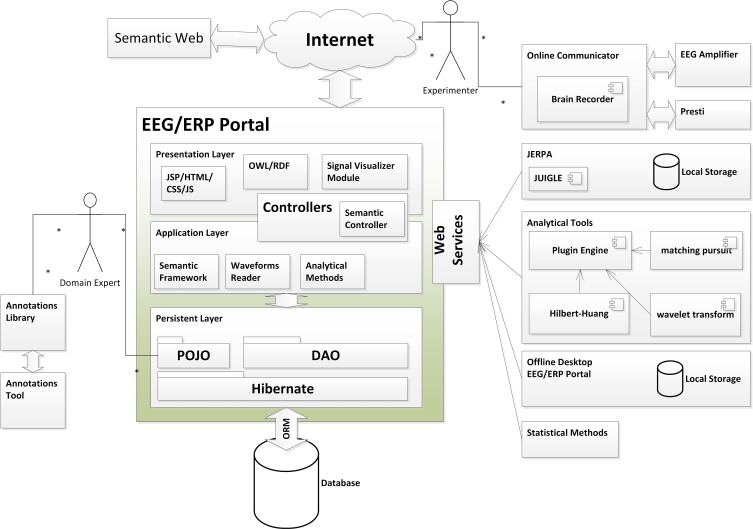
**Overall architecture (Jezek et al., 2013b)**. ^*^Means “many” (0-n occurrences) relationship.

The overall architecture of the software and hardware infrastructure for research in electrophysiology comes from the set of the main activities performed by researchers during electrophysiological experiments. First, hypotheses and design of protocols for specific experiments are done. Then experiments are performed according to defined scenarios (protocols) and data and related metadata are collected. During the experiment the EEG signal obtained from the scalp of the tested subject is synchronized with presented stimuli. Second, the data are analyzed using various processing methods. Then, the data are interpreted and the results are published. The biggest obstacles for science are the following: since data are not well-described, conclusions and interpretations cannot be later reproduced or verified. The methods used for data analysis are lost or their detailed parameters are not later traceable. To solve these difficulties, initiatives as (Teeters et al., 2008) have been established to support experimental data sharing. The development of the complex infrastructure for experiments in the EEG/ERP domain contributes to international efforts in the electrophysiology domain. An overall architecture of this infrastructure is shown in Figure 1.

The basic aim of this infrastructure is to increase effectiveness and efficiency of scientific research in the field. The central point of the infrastructure, the EEG/ERP Portal (EEG/ERP Portal, 2013), is a service providing interface to human users and software tools. The main features of this service include long-term and sustainable storage of data and related metadata collected from experiments, various methods and workflows for data processing, and sharing of data, documents, methods and workflows in groups.

An initial idea of this infrastructure was particularly described in Jezek et al. (2013b). Besides a classical web based interface intended for human readers several communication interfaces for external tools have been implemented. Standalone tools including JERPA, offline and mobile version of the EEG/ERP Portal, or tools for signals visualization communicate with the EEG/ERP Portal using web services. Other tools as a Semantic Framework are implemented as libraries integrated directly within the EEG/ERP Portal. A substantial part of a complex infrastructure is created by several third-party hardware devices and software tools. These devices and tools are controlled by the experimenter who interacts with the EEG/ERP Portal using a web browser on a standard computer, or using a mobile version of the EEG/ERP Portal.

### 2.3. EEG/ERP portal

The EEG/ERP Portal is a mature web-based system that enables researchers to upload, download and manage EEG/ERP experiments (data, metadata, experimental scenarios, etc.) (Jezek and Moucek, 2012b). The features of the EEG/ERP Portal also include sharing of knowledge, working in research groups, manage scientific discussions, run methods for signal processing, etc.

Different users have different roles in the system and the related level of authority. The users' credentials are required when users access the system. Individual users are grouped into self-managed groups. The user who wants to upload or download experiments has to be registered within the system and has to become a member of at least one group. On the basis of activities that the user can perform, several user roles are defined (Reader, Experimenter, Group Administrator, and Supervisor).

**Figure 2:**
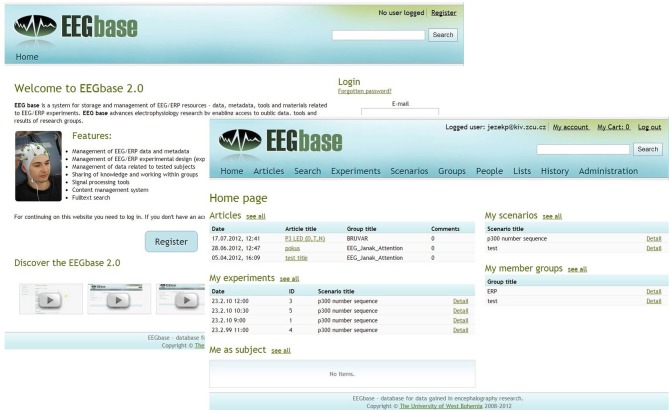
**EEG/ERP Portal Overview**. The login page and the home page of a logged user are shown. The logged user can see summarized information about his/her activities.

A simple wizard that guides the logged user through the process of adding an experiment facilitates upload of an experiment. Each experiment contains raw data supplemented by related metadata. A set of metadata which the user is instructed to fill in through the prepared forms is defined. These metadata are organized in semantic groups (experimental protocol, experimenters and tested subjects, used hardware, description of raw data, etc.) in accordance with an internal ontology initially presented in Jezek and Moucek (2011b). The experimenter can also decide if the experiment is private or public. Public experiments are downloadable for all registered users (without personal data of tested subjects), while private experiments are downloadable only within the experimenter's group. The functionality that includes possibility to associate experiments into experimental packages is in development. Individual packages can have a different access level. Experiments in these packages can be managed in bulk. The overall preview of the EEG/ERP Portal is shown in Figure 2.

Since the EEG/ERP Portal cooperates with a set of associated submodules, several communication interfaces for external tools have been designed and implemented. The tools can be divided into two groups. The first group includes tools accessible through an internet browser. These tools are implemented as stand-alone libraries integrated within the EEG/ERP Portal directly. The most important tool is the Semantic Framework (Jezek and Moucek, 2012a). The aim of the Semantic Framework is to provide experimental metadata in the semantic web languages and technologies (RDF, OWL). Data expressed in these languages and technologies are readable by semantic reasoners.

The second group of tools includes desktop or web-based tools that run locally on the user's computer. These tools access data in the EEG/ERP Portal and these data are then processed locally. The Electroencephalography Data Processor (EEG Data Processor) (Jezek and Moucek, 2013b) is a system for running methods for signal processing that enables a remote processing of data from the EEG/ERP Portal. The methods for signal processing are installed as plug-ins. Data can be uploaded directly using the web interface, or through the web service endpoint.

### 2.4. Data models and ontologies

The data model of the EEG/ERP Portal was first proposed in 2008 and since then has changed several times. Currently the core ERA model contains more than 70 tables. However, the flexibility of the data model and possibility to share data within community are still more important in recent years. Then, the main goal of data model improvement and ontology development is to increase data sharing abilities of the Portal. Currently, ontologies have become not only recommended but even required domain descriptions (e.g., NIF third level registration requires an ontological description). Besides existing projects a new Ontology for describing Experimental Neurophysiology (OEN) (Bruha et al., 2013) is being developed. The group working on the development of this ontology was formed from the members of the following initiatives:

- EEG/ERP Portal (EEGBase) (http://eegdatabase.kiv.zcu.cz/home.html)
- G-Node (www.g-node.org)
- INCF Task Force on standards for sharing of electrophysiology data (http://www.incf.org/programs/datasharing/electrophysiology-task-force)
- NIF (www.neuinfo.org)
- Neuroelectro.org (www.neurolectro.org)

The group follows the best practices for creating ontologies, for example, it cooperates with community of researchers who design and create ontologies, uses existing data formats and repositories (odML, HDF5), and reuses existing resources (terms, ontologies - NEMO, OBI). For the general description of experimental neurophysiology, the terms from ontologies NEMO and OBI are relevant. However, the set of the domain terms is still not complete in these ontologies (information stored in the EEG/ERP Portal cannot be fully described by these ontologies) and OEN will be finally an extension of OBI (e.g., the granularity of OBI for devices and related information will be extended).

**Figure 3:**
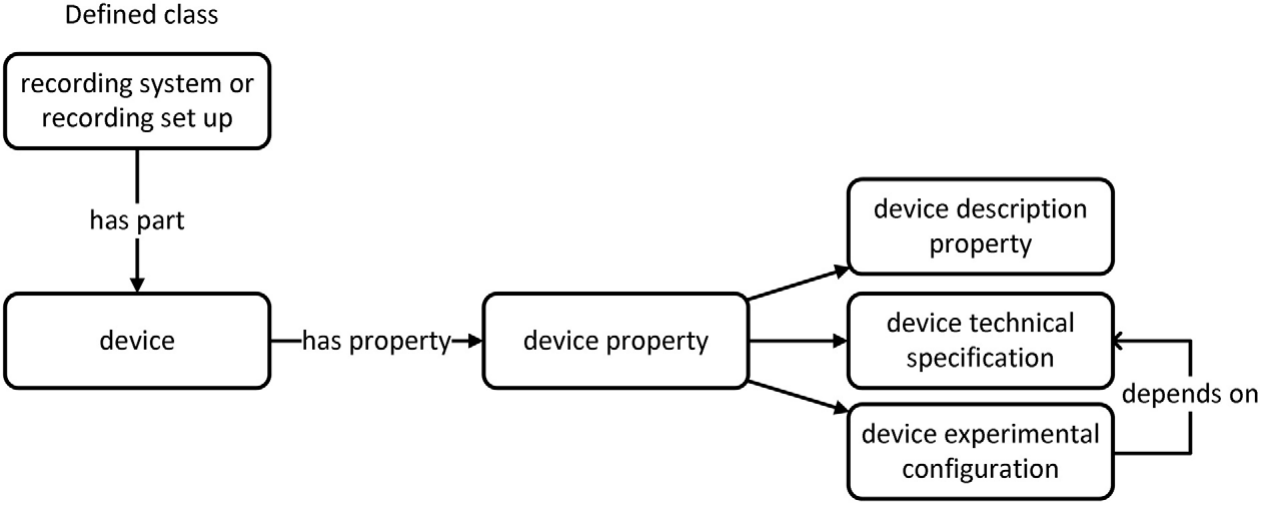
**Device knowledge model (Bruha et al., 2013)**.

Currently, the development of OEN has been separated into two branches. The first branch deals with structured terminology to annotate experimental metadata (e.g., devices or methods); the second branch deals with structured terminology to annotate experimental data (e.g., action potential). The knowledge model of 'device branch' is shown in Figure 3.

Terminologies within OEN have been primarily developed in the odML format. Subsequently, an OWL file has been constructed aided by Ontofox (Xiang et al., 2010). The current developer's version of OEN is available at https://github.com/G-Node/OEN.

**Figure 4:**
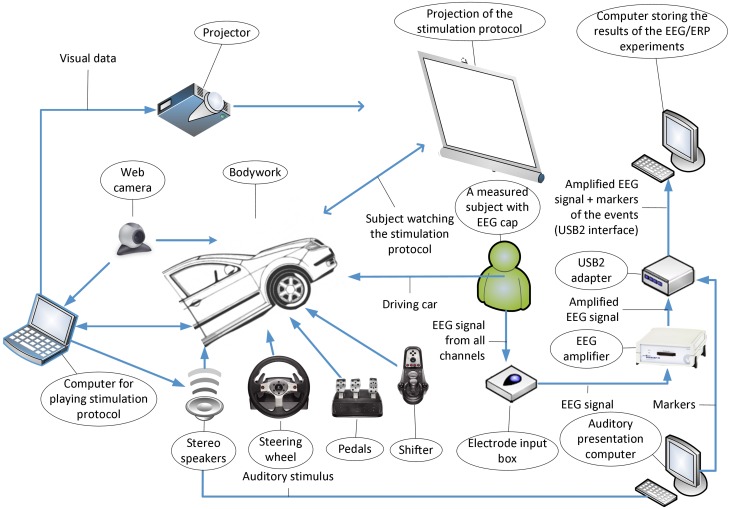
**Drivers attention experimental setup (Bruha et al., 2013)**.

To define the terms and to create the 'device branch' of OEN the following schemas that describe the elements of experimental setups and interactions between these elements were used: the experimental setup for investigation of driver's attention (Figure 4), experimental setup for performing traditional oddball experiment, and experimental setup for investigation of mouse visual cortex. Based on these schemas the preliminary knowledge model representing the experimental setup has been constructed. This model can be used to annotate the EEG/ERP Portal.

### 2.5. Signal processing methods

A subset of signal processing methods suitable for ERP waveforms detection and classification, alternatively for clustering the feature vectors extracted from ERP signal was investigated, modified and implemented by the members of our research group. These methods can be run as web services within the Electroencephalography Data Processor.

Hilbert-Huang transform [HHT, see (Huang et al., 1998) for details] is a signal processing method designed especially for non-linear non-stationary signals. It consists of empirical mode decomposition (EMD) and Hilbert spectral analysis (HSA). During the process called sifting EMD decomposes signal to intrinsic mode functions (IMF) and residue. HSA computes an analytical signal from IMF and then analytical signal instantaneous attributes. Original HHT algorithm is not fully suitable for EEG signal processing, because EEG is a quasi-stationary signal. In Ciniburk (2011) we introduced the way HHT can be used for ERP waveforms detection. In Prokop (2013) we introduced particular improvements of the classifiers for ERP waveform detection that work with HHT results. Currently, the classification reliability of the ERP detection by the modified HHT is comparable with continuous wavelet transform and matching pursuit algorithm (see Ciniburk, 2011).

The traditional matching pursuit algorithm (MP) as proposed by Mallat and Zhang (1993) is suitable for EEG/ERP signal processing because the subset of atoms from the Gabor base is correlated with ERP components Benar et al. (2007). However, the computational complexity of its brute force implementation is challenging for on-line calculations. One of the most promising implementations (Ferrando et al., 2002) is based on restricting the combinations of Gabor parameters that need to be used for scalar product calculations, an approximation of the original signal. We showed that MP with GD can be used as a suitable preprocessing method for the task of ERP detection based on a classifier which works with Vigner-Wille transform of MP result. We identified a few issues which led to false positive/negative ERP waveform detection results and solved some of them. In Rondik (2010) we based the classification on correlation between a model of ERP waveform and a signal reconstructed from significant atoms. We also introduced solution for the case if an ERP component is approximated by two or more atoms.

Various algorithms were investigated regarding their benefits for off-line BCI systems, and ERP component detection. For the P300 BCIs purposes, a multi-layer perceptron (MLP) was used to classify the features obtained using matching pursuit (Vareka, 2012) and discrete wavelet transform (Vareka and Mautner, 2013). For the MLP design, one hidden layer was used and the number of neurons was optimized using a validation dataset. The main goal of the research was to evaluate if the multi-layer perceptron is suitable for the P300 detection and to find the architectures and training algorithms that perform comparably well for this task. We were able to prove on the off-line dataset that the trained MLP neural network with the architecture described in Vareka (2012) is able to detect the P300 component as successfully as other state-of-the-art classification approaches.

Neural networks have also been used to cluster the feature vectors that were extracted from the ERP signal. The ERP signal reflects not only ERP components, but also artifacts and background EEG activity. The objective was to analyze the signal and to try to separate different waveforms without using reliable but computationally complex Independent Component Analysis as proposed in Makeig et al. (1997). Furthermore, since the latency of ERP components may vary for different subjects, or stimulation protocols (Luck, 2005), the method can also be used to cluster the feature vectors assigned to a specific ERP component to further analyze how the component might be affected by external factors or disease. In Vareka and Mautner (2012), the features were extracted from the signal using matching pursuit (Mallat and Zhang, 1993). The ART 2 neural network (Carpenter and Grossberg, 1987) was used to cluster the ERP features. The optimal adjusting parameters for the ART 2 neural network were found. As a result, the traditional ART 2 network was proven to be useful for our experiments. The proposed architecture is described in more detail in Vareka and Mautner (2012).

### 2.6. Workflows

Data obtained from electrophysiological experiments are analyzed using various preprocessing and processing methods, some of them are described in Section 2.5. However, there is usually a need to use more than one method for analysis of the EEG/ERP signal. Therefore, we provide an opportunity to define workflows for complex analysis of experimental data. In our domain, a workflow includes a complex set of analytic methods that process experimental data sequentially or in parallel. It is organized as a tree structure, where each branch of the tree has the same meaning as a pipe in Linux; an output of the method serves as an input of the next method. The whole workflow process is divided into simple tasks—work steps. The work step includes one analytic method and requires following information (Mrvec, 2013):

- Name - identification of a work step
- Format - used data format as an input to a method
- Store - a decision, whether a result from a previous method should be stored
- Data - an input to a method e.g., data files or a name of previous workstep
- Method - a name of the used method with its parameters

A work unit representing a sequential workflow is composed of work steps. This solution allows creating more workflows with the same or different input data.

### 2.7. Mobile and offline portals

The advantage of the EEG/ERP Portal is its accessibility from all computers connected to the Internet. Such solution is sufficient for collecting experiments performed in the laboratory. On the other hand, situations when a standard computer is not available are frequent. It includes situations when experiments are conducted outside the laboratory using a portable measuring device. In this case paper forms that are backward transferred to a central database are used. This process can be cumbersome, confusing, and error-prone. In addition, when data are collected electronically, they can be validated at the time of collection. It protects making logical errors or notational problems, and ensures that the required forms are complete.

Another use case is a situation when a researcher discusses experimental results with colleagues at workshops. He/she probably does not have desired data on hand. A mobile device used in everyday life, such as a mobile phone or tablet seems to be a practical solution for presenting experimental data.

**Figure 5:**
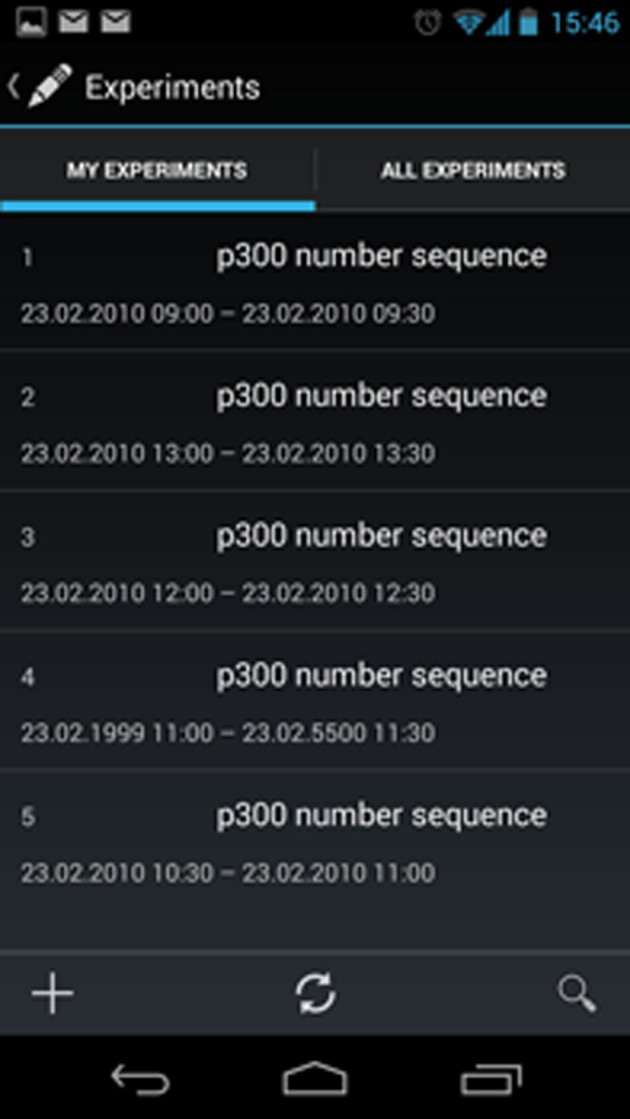
**The mobile system preview**. The print screen shows a list of available scenarios. When a user clicks to a specific scenario, a detail piece of information appears. The top bar allows users to add a new scenario (using “+” button), search existing items (using magnifying glass), or refresh the list.

With regards to the mentioned needs and difficulties a system for collecting experimental data/metadata running on mobile devices has been developed. The aim of this system is to serve as a mobile version of the EEG/ERP Portal. This mobile portal provides similar functionality as the common EEG/ERP Portal. The data from this device are synchronized with the data stored in the EEG/ERP Portal. This solution significantly reduces the usage of paper forms during experimenting. A preview of the mobile EEG/ERP Portal is shown in Figure 5.

**Figure 6:**
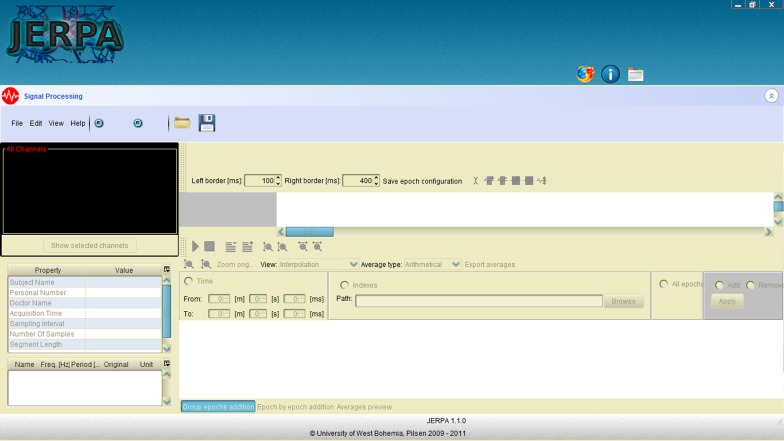
**JERPA overview**.

An offline EEG/ERP Portal is a next useful system developed outside the EEG/ERP Portal. It is designed to be installed on computers or laptops without a permanent internet connection. The offline EEG/ERP Portal became a part of the JERPA software tool (Jezek and Moucek, 2011a)—a desktop system for running signal processing methods and signal visualization. This system contains a powerful plug-in engine that enables installing signal processing methods as plug-ins. A server-client approach is used. A module that ensures an online access to experimental data stored in the EEG/ERP Portal implements a web service client. The EEG/ERP Portal represents the server side. Downloaded data are stored in an embedded database and they are available when the system gets offline. When a new experiment is added, it is synchronized with the EEG/ERP Portal when the system returns online. The stored data are ready to be processed by installed methods. An overview of the JERPA system is shown in Figure 6.

### 2.8. Programmable hardware stimulator

A programmable hardware stimulator was designed and developed for EEG/ERP experiments performed in our neuroinformatics laboratory. The main idea was to have a portable device which was very easy to use and did not require another hardware device (PC or laptop) needed for experiments. In addition, we also wanted to compare the results (e.g., timings and delays) from experiments in which a software stimulator had been used with the results from experiments in which a hardware stimulation device had been used.

**Figure 7:**
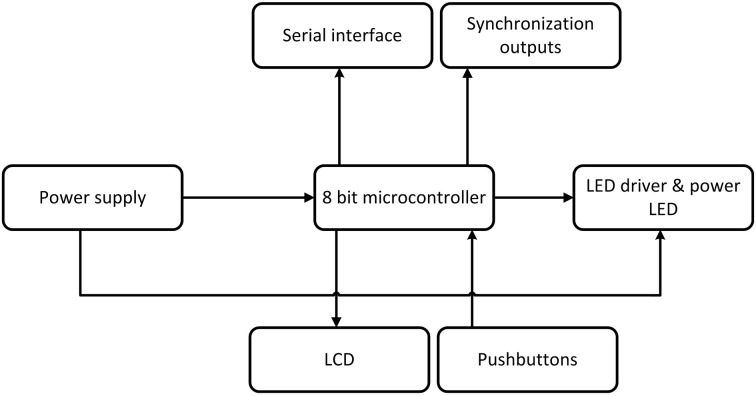
**Block diagram of the stimulator**.

To design and construct a first prototype we used a simple 8-bit microcontroller with an interrupt based firmware which works as a timed LED driver. The basic structure can be seen in the block diagram in Figure 7. This implementation was expanded step by step with different features like LED brightness, scalable distribution schemas, and new experiments predefinitions.

The finalized version provides a fully programmable setting of stimuli parameters in two scenarios. The first scenario is an implementation of the oddball protocol and the second scenario enables multi-source frequency stimulation. A simple GUI and serial port communication protocol were implemented.

At the moment we are working on a new version of the stimulator based on experience gained by using this prototype during experiments. It will provide a more comfortable user interface, a broader opportunity to set parameters for EEG/ERP experiments, and new possibilities in stimuli generation for auditory stimulation protocols.

## 3. Results

This section provides information about the current state of the proposed infrastructure and some implementation details of the parts of the infrastructure described in Section 2.

### 3.1. EEG/ERP portal

The EEG/ERP portal is a central point of the complex architecture presented. It is a powerful tool intended to serve to a wide researcher's community. It facilitates management of experimental data; provide an interface for accessing them, and due to well-defined ontology it significantly helps in interpretation of experimental data. The portal interface is suitable not only for human readers who access it using a web browser, but due to an advanced web service endpoint it can be easily integrated with complementary tools. Such infrastructure is ready to be used not only in our laboratory but also by other interested researchers.

A core of the EEG/ERP Portal creates the Spring framework (Walls, 2011) that provides a comprehensive programing and configuration model for Java-based enterprise applications. The data layer of the EEG/ERP Portal uses the Oracle database system (Greenwald et al., 2007). The Hibernate framework (Bauer and King, 2006) ensures persistence of data transferred between the database and a Java-based application layer. The presentation layer is created by the Apache Wicket framework (Dashorst and Hillenius, 2008) that facilitates implementation by a system of reusable components written with plain Java and HTML. The privacy of stored data and integration with social networks as LinkedIn or Facebook are ensured by subcomponents of Spring: the Spring security framework and the Spring social framework. The Spring security framework uses a XML-based configuration for an authorized access to individual web pages. The Spring social framework provides a unified API to access various social networks.

The main integrated tool, the Semantic Framework (described in Section 2.3), is being developed as a single library. From the user's perspective, it is used as a black box with the input in the form of a collection of Java persistent objects and the output in the form of an ontology document. The ontology document can be serialized into several supported syntaxes [currently RDF/XML (W3C Consortium, 2004), OWL/XML (Motik and Patel-Schneider, 2008), Turtle (W3C Consortium, 2008), and abbreviated OWL/XML formats are supported]. The Semantic Framework is controlled by a build-in timer. The timer calls the Semantic Framework API in regular intervals. The API generates the ontology document from the stored experiments and saves this document to a temporary file. When any document request appears, the temporary file containing the current set of stored experiments is immediately available.

External tools work independently of the EEG/ERP Portal. The EEG/ERP Portal provides an interface for accessing stored experiments using Web Services technology; RESTfull (Richardson and Ruby, 2007) and SOAP (Snell et al., 2002) web services are used. These web services are secured by user credentials and provide several methods to access user's experiments including raw data, metadata and experimental scenarios. An interested client only implements a web service client. Several tools presented in this paper such as the offline EEG/ERP Portal or mobile EEG/ERP Portal implement the web service client to access experimental data and so prove the validity of the approach presented.

### 3.2. Data models and ontologies

The EEG/ERP Portal was registered as a neuroscience resource within the NIF at the level 2.5; this level allows users direct access to the services implemented within the EEG/ERP Portal. Privacy and security of the stored data are guaranteed by data and metadata anonymization. Currently, the data about tested subjects, raw experimental data, data related to used hardware, experimental protocol (scenario), and other experimental parameters (e.g., length of recording) are accessible via NIF (Bruha and Moucek, 2012). The Portal is intended to be registered at the 3rd level of NIF registration schema. This step will be accompanied by the annotation of the Portal data and metadata using the OEN ontology.

**Figure 8:**
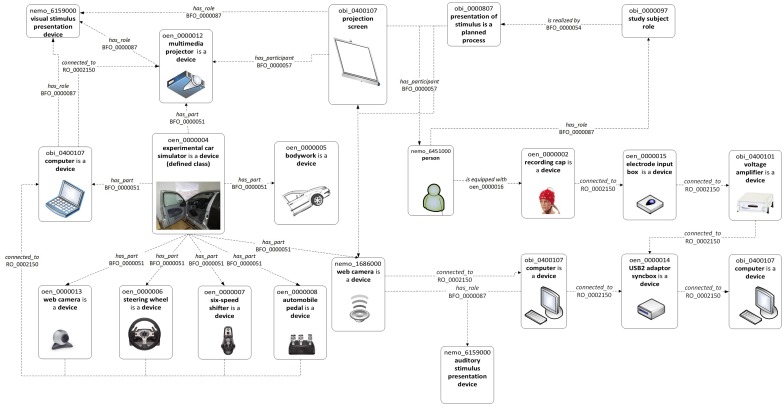
**EEG/ERP Portal device knowledge model (Bruha et al., 2013)**.

Figure 8 shows how to describe the experimental set-up using terms (labels/names in bold) from OBI (obi_xxxxxxx), NEMO (NEMO_xxxxxx), OEN (oen_xxxxxxx) and relations (dashed arrow, label, and id) (Bruha et al., 2013).

### 3.3. Signal processing methods

**Figure 9:**
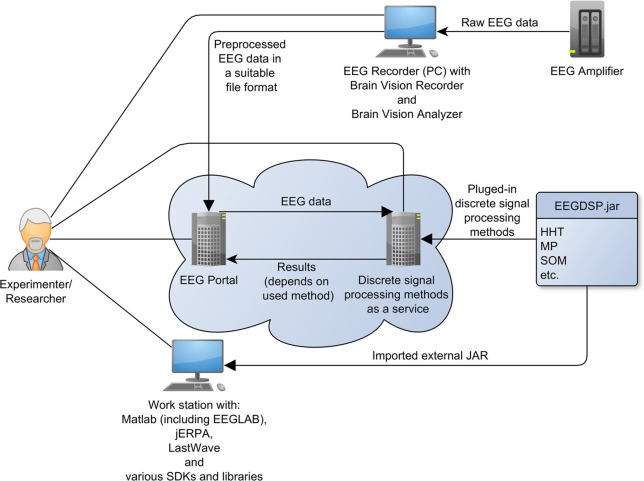
**Signal processing within the infrastructure**.

From researchers' point of view, our signal processing methods infrastructure consists of the following commercial elements: MATLAB (including EEGLAB Delorme and Makeig, 2004 plug-in) MATLAB (2012), the BrainVision Recorder and the BrainVision Analyzer applications (BrainProducts, 2012). Furthermore, the following elements of our infrastructure are freely available: Lastwave (Bacry, 2012), JERPA (Jezek and Moucek, 2011a), the EEG/ERP Portal, the EEGDSP java library (described below), and the EEG Data Processor (Jezek and Moucek, 2013b). Figure 9 shows this infrastructure including relationships between the elements.

Researchers can access and work with all elements except the EEG amplifier and the EEGDSP.jar library. The reason is that both of them need an interface that provides them functionality. In case of the EEG amplifier, the interface is the Brain Vision Recorder. In case of the EEGDSP.jar library, the interface is the EEG Data Processor or all programing languages which can call external Java libraries.

The EEGDSP library was implemented in the Java language and includes basic methods and approaches for discrete signal processing: wavelet transform, matching pursuit algorithm, fast ICA, FIR filters (low pass, high pass, band pass, band reject), window functions (BarlettHann, Barlett, BlackmanHarris, BlackmanNuttalll, Blackman, Bohman, Cosine, FlatTop, Gauss, Hamming, Hanning, Kaiser, Lonczos, Nuttall, Parzen, Rectangular, Triangular, and Tukey), and Hilbert-Huang transform. The implementation of the Hilbert-Huang transform uses the modified HHT algorithm (Section 2.5) to detect ERP components in the EEG signal (there was no free or commercial library with implemented modified HHT before). To facilitate the usage of the discrete signal processing methods by researchers and services, the application Electroencephalography Data Processor (Section 2.3) was implemented. The data processing feature is powered by the EEGDSP library.

### 3.4. Worflows

The workflow management system is currently under development. Any workflow is described by an XML file. We chose the XML format since it is independent of the used platform and programming language. An example of the workflow description is given below (Mrvec, 2013).


<?xml version="1.0" encoding="UTF-8"?>
<workflow name="Workflow">
 <workunit name="Experiment1">
   <workstep name="SimpleFile" format="KIV_FORMAT"
                   store="false">
     <data>data1.eeg</data>
     <data>data1.vhdr</data>
     <method params="01,100,Cz,FAST_DAUBECHIES_2">
                     DWTPlugin-1.0.0</method>
   </workstep>
   <workstep name="SimpleDouble" format="DOUBLE_FORMAT"
                   store="true">
     <data>Experiment1_SimpleFile</data>
     <method params="01,1000,Cz,COMPLEX_GAUSSIAN,
                     1,1,1,14000,14000">
     CWTPlugin-1.0.0
     </method>
   </workstep>
</workunit>
<workunit name="Experiment2">
   <workstep name="SimpleFile" format="KIV_FORMAT"
                   store="true">
     <data>data2.eeg</data>
     <data>data2.vhdr</data>
     <method params="01,100,Cz,FAST_DAUBECHIES_2">
                     DWTPlugin-1.0.0</method>
   </workstep>
</workunit>
</workflow>


This XML file is generated while the user creates a workflow (he/she selects methods, defines values of their parameters, and puts them into analytic pipelines). When the user finishes his/her workflow, the XML file is transferred to a processing unit that is responsible for parsing the file and calling required methods. This approach allows changing a source of analytic methods (e.g., EEG Data Processor, Matlab scripts, or local libraries) without changing a generation process of the descriptive file. It is only necessary to change the processing unit and a graphic user interface.

Since the used analytic methods have various input/output parameter types, it is necessary to ensure their compatibility in sequential workflows. It means that the output from a previous method and the input to a next method must match. We ensured the syntactic compatibility by comparison of input/output parameters types. Each used method has a definition of input/output parameters by the XML file attached to the methods.

However, for well-designed workflows, ensuring syntactic compatibility is a necessary, but a single step. The used methods have to be also connected correctly in terms of their semantics (if their connection makes sense or not). Therefore, we will focus on designing the semantic compatibility in the future.

### 3.5. Mobile and offline portals

Because of difficulties with unavailability of standard computers in many environments we developed a mobile version of the EEG/ERP Portal that is able to fully substitute the EEG/ERP Portal when experiments are performed outside the laboratory. This solution profits from rising popularity of mobile devices such as tablets or mobile phones. The presented implementation can be extended to enable work with other electrophysiological databases. It will result in a domain independent system using a customizable user layout.

When the user works on a portable device as a laptop in environments when the internet connection is not available (as hospitals or other institutions outside the laboratory), the offline version of the EEG/ERP Portal is available.

From the implementation point of view, the mobile EEG/ERP Portal contains a set of forms where the user can fill in metadata describing an experiment. The set of metadata is equivalent to the set of metadata that the user can fill in the common EEG/ERP Portal. The communication of both the mobile EEG/ERP Portal and web EEG/ERP Portal is ensured using RESTfull web services. Server-client architecture is used. The server part is implemented in the EEG/ERP Portal. The server provides access to the database and sends data to the client implemented inside the mobile device. The communication between the server and client is secured using the SSL protocol. User credentials are required; the EEG/ERP Portal user account is used to verify the client (Jezek and Moucek, 2013a).

### 3.6. Programmable hardware stimulator

**Figure 10:**
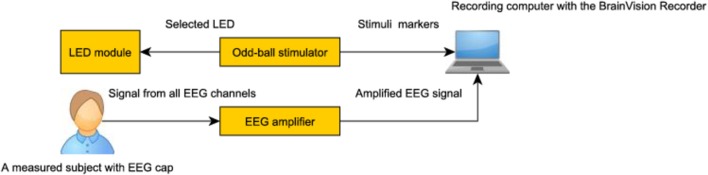
**Experimental usage of the programmable hardware stimulator**.

The hardware stimulator described in Section 2.8 was designed and tested for elicitation of the visual P3 component. The arrangement of a typical experiment and connection of the developed stimulator to the ERP recording system are presented in Figure 10. The standard oddball task was used to verify the functionality of the proposed hardware stimulator. In this task, red and green LEDs representing non-target and target stimuli were randomly switched on and off for a period of 0.5 s. The probability of the target stimuli (i.e., the green LED was switched on) was set up to 0.2. Currently, the hardware stimulator was successfully used for stimulation of 15 tested subjects.

### 3.7. Software tools licence information

The tools developed at our department, including the EEG/ERP Portal, the mobile EEG/ERP Portal and the Semantic Framework, are distributed under the Apache License 2.0. The EEGDSP library, JERPA, and the EEG Data processor are distributed under the GNU General Public License v.3. All the tools mentioned above are hosted in GitHub repositories. The EEG/ERP Portal is available under the INCF group at https://github.com/INCF/eeg-database. The EEG/ERP Portal is running on http://eegdatabase.kiv.zcu.cz. The following libraries, including the EEGDSP library, JERPA, the mobile EEG/ERP Portal and the Semantic Framework are hosted under the neuroinformatics group and available at https://github.com/NEUROINFORMATICS-GROUP-FAV-KIV-ZCU.

## 4. Discussion

There are a lot of difficulties with collection, storage, management and interpretation of electrophysiological experimental data and metadata. Any complex software and hardware infrastructure supporting research and researchers in this field can contribute to efficiency and effectiveness of researchers' work.

This paper shortly introduced the infrastructure for research in electrophysiology that has been continuously built in the neuroinformatics laboratory at the Department of Computer Science and Engineering, University of West Bohemia. Over time the parts of the infrastructure have become more general with the potential to serve to the wider scientific community. Of course, there are still many infrastructural parts that need to be changed, finished or even only properly designed.

The central point of the described infrastructure, the EEG/ERP Portal, serves as a data management tool that provides services for other supplementary tools. Because the relational database is currently used as persistence storage, we are facing difficulties with storing heterogeneous experimental data. Our next step leads to the usage of a NoSQL database instead of the relational one. Currently we test Elasticsearch for its full text search capabilities. The future direction is to provide a domain independent metadata structure that enables to store various experimental data from laboratories.

Because some experiments are conducted outside the laboratory, the mobile version of the EEG/ERP Portal was presented. In response to positive feedbacks the next significant step is to provide an extension of this system independent of the EEG/ERP Portal. Such extended system will communicate with other domain independent electrophysiological databases. The layout of this system will be generated automatically as proposed in Jezek et al. (2013a). odML as a unified metadata format will ensure a server-client data transfer. Using a NoSQL database also means to modify the supplementary tools, the offline EEG/ERP Portal, and the JERPA system.

The ontology development was first focused on the experimental data and metadata stored in the EEG/ERP Portal. Currently, the emerging OEN ontology is not a specific ontology describing just the data and metadata stored in the EEG/ERP Portal; its concept is open for any neurophysiological needs. Nevertheless, the EEG/ERP Portal will be the first use case fully described by this ontology. This will also help to fulfil NIF requirements for the registration at the 3rd level of the NIF portal. Then the data and metadata from the EEG/ERP Portal will be fully accessible to other communities and research groups via the NIF interface.

Various tools for EEG signal processing have been used by our research group. Certainly, researchers would benefit from a possibility of having all methods at the same place. The EEG Data Processor seems to be an appropriate solution. However, there are some issues to be solved. We are not able to implement all methods that researchers need. Therefore, we plan, according to the best principles in software engineering, to implement a common interface which encloses the implemented methods and gives researchers a unified way to use them on a source code level; then it is easy to include custom methods into the EEGDSP implementation. In the medium term, we will focus on distributed computing as well as on load balancing.

For a complex analysis, more methods are combined sequentially or in parallel; the workflows are created. The presented prototype of the workflow management system is still under development. The proposed description of workflows and the used XML language bring the independence of the methods used and the programming language in which they are written. In the future, the workflow system will be integrated into the EEG/ERP Portal.

The programmable hardware stimulator is applicable to a variety of experiments. The modular design of the firmware allows users to modify stimulation protocols according to experimenters' requirements easily. In the near future, a miniaturized stimulator using 32bit MCU will be developed. Moreover, this stimulator will allow users to apply a larger set of stimulation methods and thus it can be used in a larger number of experimental protocols (e.g., our research group plans to use it for BCI experiments and for the stimulation of the mouse brain).

We are aware that the current state of the infrastructure is intended for storing, maintenance and analysis of EEG/ERP waveforms and related metadata. On the other hand, long-term work on this initial infrastructure has helped the research group to understand heterogeneity of neurophysiological data, limitations of the proposed methodology and also limitations of used technologies. The future work of the research group thus includes changes in methodological concepts (e.g., usage of international standards for data formats and ontologies or definition of a wider collection of use cases in neurophysiology) and technologies (e.g., technological solutions supporting more flexible organization of data). On the other hand, there is also the danger that the proposed infrastructure could be too general. It results in difficult implementation, configuration, and too complex and demanding user interface. Aware of both the difficulties and dangers, too high specialization and/or too high abstraction of the system, the research group intends to continuously improve the existing infrastructure for specific purposes. In parallel, it plans to extend the ability of the existing infrastructure to store and process a larger variety of neurophysiological data by following the international standardization efforts and by respecting the needs of researchers. Some of these methodological and technological steps (OEN ontology, odML format, NoSQL database) are already described in this article.

### 4.1. Data sharing

Our catalog server connected to INCF Dataspace and a node for eeg/erp domain (a subnode of the catalog server) were established. Specifically, the node named “cz.zcu.eeg” contains the collection named “experiments” with a set of subcollections grouped according to experimental scenarios. These subcollections contain experimental data divided into individual sessions. Metadata are stored in CSV files. The node server is synchronized with the data in the EEG/ERP Portal in regular intervals using an implemented timer. It ensures availability of up-to-date experimental data. The data stored in the EEG/ERP Portal are also shared via NIF.

### Conflict of interest statement

The authors declare that the research was conducted in the absence of any commercial or financial relationships that could be construed as a potential conflict of interest.
