# Corrigendum: Software and hardware infrastructure for research in electrophysiology

**DOI:** 10.3389/fninf.2014.00075

**Published:** 2014-09-05

**Authors:** Roman Mouček

**Affiliations:** ^1^Department of Computer Science and Engineering, University of West BohemiaPlzen, Czech Republic; ^2^New Technologies for the Information Society, University of West BohemiaPlzen, Czech Republic

**Keywords:** electrophysiology, event related potentials, infrastructure, neuroinformatics, workflow, portal, signal processing methods, stimulator

This article adds Yann Le Franc as a co-author of the technology report article “Software and Hardware Infrastructure for Research in Electrophysiology,” describes his individual contribution to the article, and presents changes in two paragraphs in Section 2.4, where an additional reference is also provided. Moreover, the members from the OEN group who work and cooperate on building OEN are personally acknowledged.

**Co-author:** Yann Le Franc

**Affiliation:** e-Science Data Factory S.A.S.U., Paris, France; Ludwig-Maximilians-Universität München, Planegg-Martinsried, Germany; University of Antwerp, Antwerpen, Belgium

**Authors' Contributions**

Yann Le Franc contributed to the **Figures 3,4** and **8** and to the work on the Ontology for describing Experimental Neurophysiology (OEN).

**Section 2.4**

Old version:

The group follows the best practices for creating ontologies, for example, it cooperates with community of researchers who design and create ontologies, uses existing data formats and repositories (odML, HDF5), and reuses existing resources (terms, ontologies—NEMO, OBI). For the general description of experimental neurophysiology, the terms from ontologies NEMO and OBI are relevant. However, the set of the domain terms is still not complete in these ontologies (information stored in the EEG/ERP Portal cannot be fully described by these ontologies) and OEN will be finally an extension of OBI (e.g., the granularity of OBI for devices and related information will be extended).

New version:

The group follows the best practices for creating ontologies, for example, it cooperates with community of researchers who design and create ontologies, uses existing data formats and repositories (odML, HDF5), and reuses existing resources (terms, ontologies—NEMO, OBI). For the general description of experimental neurophysiology, the terms from ontologies NEMO and OBI are relevant. However, the set of the domain terms needed to describe the information stored in the EEG/ERP Portal is not yet complete in these ontologies. OEN aims at defining these missing terms and at term, should be used to propose an extension of OBI's neurophysiology model (e.g., the granularity of OBI for devices and related information will be extended).

Old version:

Terminologies within OEN have been primarily developed in the odML format. Subsequently, an OWL file has been constructed aided by Ontofox (Xiang et al., 2010). The current developer's version of OEN is available at https://github.com/G-Node/OEN.

New version:

The OEN device branch development is based on the odML terminology (Grewe et al., 2011), concepts gathered by the Neuroscience Information Framework (NIF) and concepts used in the EEGBase data model to describe setups and setup configurations. The gathered terms are currently mapped with the aforementioned ontologies. Subsequently, an OWL file has been constructed to contain OEN terms and the mapped terms. Existing terms in other ontologies will be imported using the MIREOT approach (Courtot et al., 2011), aided by Ontofox (Xiang et al., 2010). The current developer's version of OEN is available at https://github.com/G-Node/OEN.

## Conflict of interest statement

The author declares that the research was conducted in the absence of any commercial or financial relationships that could be construed as a potential conflict of interest.
